# The Study of Legal Medicine

**Published:** 1911-09-02

**Authors:** William A. Brend

**Affiliations:** of the Inner Temple, Barrister-at-Law.


### THE STUDY OF LEGAL MEDICINE.

By WILLIAM A. BREND, M.A., M.B., B.Sc., of the Inner Temple, Barrister-at-Law.

This subject may be best considered from the point of view of the medical student and from that of the qualified medical practitioner separately.

To the average medical student only those applications of law to medicine which are likely to be the subject of question in an examination are of interest. For the time being the syllabus laid down for the course in "Forensic Medicine" or "Medical Jurisprudence" contains all that he wishes to know. It may be impressed upon him that much of the law regarding medical practice and practitioners is not included in this syllabus, and that sooner or later he will have to acquire some of it at least, or suffer from the want of it; yet his impulse as a student will certainly be to defer this study until his post-graduate days. Nor can this attitude be seriously deprecated considering the wide extent of the medical curriculum. The danger is that what is merely to be deferred may never be done at all.

### Medical Jurisprudence.

Forensic medicine is usually defined as "the application of medical sciences to the purposes of the law," and the standard text-books on the subject in selecting the material to be considered usually adhere fairly strictly to this definition. It is in the elucidation of deaths from violence that the law calls most frequently for the aid of medical science, and accordingly the bulk of the subject is a consideration of the effects produced on the body by different forms of violence. Matters involving the sexual functions, and the requirements of the law in regard to insanity, make up most of the rest. Certain subjects which scarcely conform to the above definition and are of little medical interest—as, for example, anthropometry and the study of finger-prints—are regarded as within the scope of medical jurisprudence, while other important medico-legal subjects are excluded. Every medical man should have some idea of the origin, constitution, and functions of the General Medical Council. Yet few students could answer satisfactorily a question of this nature, and if asked in an examination in forensic medicine it would probably be looked upon as outside the syllabus and unfair. It is not dealt with in the ordinary text-books on the subject.

### Theoretical and Practical Work.

The student will not find forensic medicine one of his most difficult branches of study. The examination is usually combined with that in public health, and the standard required is not so high as that in other subjects. Medical jurisprudence is practically always looked upon as a branch of medicine and dealt with from the medical standpoint. The leading text-books are written or edited by medical men, and the examinations follow the same lines. From the examination point of view, therefore, the student is not seriously prejudiced by the fact that the statements of law in some of these books are inaccurate or incomplete, though if he relies upon them later when he is in practice, he may make this discovery to his cost. There is no examination in practical work, and, except for a little chemical testing for poisons, no practical instruction in medical jurisprudence is given. In this matter the medical schools might take a hint from the Scotch universities. In Edinburgh the lectures are followed by practical demonstrations, and students are permitted to visit the city mortuary and see post-mortem examinations made for medico-legal purposes. In most of the Continental cities, too, far greater facilities are offered to students for the study of medical jurisprudence than is the case in London, though there is probably no other place where there is so much wealth of material. It is a matter for regret that in England the importance attached to the study of medical jurisprudence is so small. As things are, the student will find any of the leading text-books, or attendance at a good course of lectures, quite sufficient for his purpose.

### Legal Medicine and the General Practitioner.

But from the point of view of the medical practitioner legal medicine should be regarded as including many subjects which do not fall within the definition given above. In the first place the medical man should be thoroughly conversant with the law regarding himself and his profession. He should be aware of his liabilities under various Acts of Parliament, as, for example, in connection with births, deaths, industrial or infectious diseases, etc., and he should know the privileges and protection which are conferred upon him in return by the recognition of his legal status. He should be familiar with his legal obligations towards his patients and the risks he runs by disregarding them. He should be able to perform efficiently the special services which are required from him from time to time in connection with crimes of violence, and to give satisfactorily evidence in the coroners' and other courts of law. Finally, he should be cognisant of recent or pending legislation in connection with medical matters, and capable of criticising it and helping to carry it into effect. It is not difficult to show that many medical men fail to acquire this medico-legal knowledge, and that both they and the profession suffer in consequence.

### Legal Post-mortem Examinations.

Consider, for example, the investigation of deaths from unnatural causes. As a student, the medical man probably received no instruction in his liabilities under the Registration Act, and in the proper way to fill up a death certificate, or when he should refuse to give a certificate or report a case to the coroner. Hence it happens that some cases are reported unnecessarily, while others which should certainly be reported are certified, and coroners have legitimate grounds for their not infrequent complaints of the unsatisfactory information or certificates given by medical men. Again, a medical man may be required to make a post-mortem examination previous to the holding of an inquest. It is well known that much dissatisfaction has arisen over the way in which this duty is sometimes performed by the general practitioner. Unaware of the special precautions which have to be taken in medico-legal work, he is apt to conduct the examination on the simple lines of purely pathological investigation which is made in hospital. Vital organs are left unexamined, important indications unnoticed. Care is not taken to leave the parts in a condition for further examination by an expert if necessary, and, if later a chemical analysis is required, it may be complicated, as in the Crippen case, by the presence of preservatives which have been added. So much so is this the case that it is now urged that all such work should be taken out of the hand of the general practitioner and given to pathologists specially appointed for the purpose to each of the coroners' courts. Should this be done, the practitioner is likely to suffer both in prestige and pocket. Yet if he received proper training there is no reason why the average medical man should not be quite able to perform satisfactorily the bulk of the post-mortem examinations required by the coroners. Most of them are straightforward, and those which under any circumstances require expert investigation form only a small proportion of the whole.

### The Medical Witness.

Or take again the giving of evidence. It is notorious that the medical man makes a bad witness. Here he suffers from the fact that the books he has read deal with the matter only from the medical point of view, and give no instruction in the way in which courts of law regard evidence. In the box he fails to distinguish between theory and fact, he gives the observations of others as his own, he answers more than he is asked, and thus places himself at the mercy of counsel. Worst of all, he too frequently allows the fact that he has been called by one party to the litigation to obscure his true judgment, and to lead him to display partisanship over matters which are purely questions of scientific fact. This reproach is perhaps most frequently levelled against the general practitioner, and in cases which come under the Workmen's Compensation Act, but it is not necessary to search very far to find examples of men, holders of hospital appointments, and specialists in the subject in which they are being examined, who have cut most deplorable figures in the witness-box, owing to their efforts to strain the facts to fit in with a particular hypothesis.

Part of this state of affairs is undoubtedly due to the difference in the mental habit of the doctor and the lawyer engendered by their very different trainings. Medicine is not an exact science; with the progress of discovery its principles are constantly changing and advancing, and the physician early learns that every view is provisional and liable to be modified by circumstances. The law on the other hand is exact, its fundamental principles are practically constant, and the lawyer is trained to a precision of thought and speech which he can apply to his labours in a manner impossible to the medical man. But he does not always realise this, and is apt to demand from the sister profession the same exactness he possesses himself. Yet when full allowance is made for this difference, there still remains a considerable margin of shortcoming in the medical man as a witness, and this might be met by adequate instruction in the meaning of evidence from the legal point of view and in the proper way of giving evidence. The rules of conduct for medical men which are laid down in the books on forensic medicine are of little value. There is no attempt to deal with the general principles of evidence, and much that is of importance is omitted. On the other hand, undue stress is placed upon non-essentials. Take, for example, the advice always impressed upon the medical man to avoid using medical terms and to give his evidence in popular language. This may be all very well as a general rule, but its too close observance may easily lead to confusion and difficulties. Some medical terms cannot be translated into simple language, and precision should never be sacrificed to simplicity. In a recent case in the Central Criminal Court, where the question at issue was whether a perforation of the intestine had been caused by disease or a kick, the medical witness spoke of the "lining membrane of the intestine." He meant the peritoneum, but counsel supposed he was referring to the mucous membrane, and much confusion occurred before the misapprehension was set right. The medical man should always choose those terms which express his meaning most exactly. If they require further elucidation it is the business of counsel or coroner to do this by means of supplementary questions.

### The Study of Legal Medicine.

These matters have been cited to illustrate the importance to the medical man of a knowledge of the branches of the law which specially affect himself or his profession. If further instances are required of the way in which want of this knowledge acts as a drawback, it is only necessary to look at the pages of the annual reports issued by the various medical defence societies. A fair proportion of the cases described therein show that medical men have fallen into difficulties which might easily have been avoided had they been familiar with their legal obligations and liabilities.

So far the study of legal medicine has been considered as affecting the individual medical practitioner. But it deserves to be looked at also from a wider aspect. The application of medical science to social affairs is increasing. The public health service attacks the causes of disease, and its effects are seen in the steadily falling death-rate. A system of medical inspection and treatment of school-children extends over the country, and the creation of a public medical service involving a third of the community will shortly be an accomplished fact. The teachings of pathology are being applied to the criminal and the inebriate. The upholders of eugenics, and those who see a national menace in the falling birth-rate, alike appeal to medical science for support for their proposals. In this activity the medical man is not playing that part which his knowledge and experience specially befit him to do. His influence in the national and municipal councils is small. There is at the moment, for example, not a single medical man on the London County Council, and that body has had to tackle the enormously difficult problem of providing efficient medical treatment for the school-children within its area without any inside help from the medical profession save that given by its permanent staff.

In the House of Commons there is only a handful of men with medical qualifications, and of these only three or four have had actual experience of medical practice. There has never been a medical man in the Cabinet, and only a very small number in the Government. Under these circumstances legislation dealing with medical affairs is bound to suffer. The National Insurance Bill must be regarded as still *sub judice*, but the absence of a clause in the Midwives Act providing for the payment of practitioners called in under the Act furnishes a good illustration of the way in which the interests of the profession may be neglected. The community also suffers. Largely for want of an effective medical driving force in the House of Commons, measures of importance are postponed year after year and abuses are allowed to continue unchecked. Reform in the method of death certification is long overdue. The Acts intended to protect medical men in the use of their titles and qualifications are practically dead letters, and the harm done by unqualified practice is notorious. The open sale of abortifacient drugs is a national scandal. The laws regarding food adulteration, in spite of their number and frequent amendment, are often ineffective, and something like 10 per cent. of all the samples of milk taken are found to contain the bacilli of tuberculosis. The Coroners' Act requires overhauling. Legislation is demanded to regulate the administration of anæsthetics. Venereal disease is accepted as part of the unchangeable order of nature, and the recommendations of the Royal Commission on Physical Deterioration as far back as 1904, that an exhaustive inquiry should be made into the extent of the malady and the steps that might be taken to arrest its progress, pass unheeded.

It may be asked, What has all this to do with medical jurisprudence? The answer is that these are matters involving legislation, or changes in legislation, which can only be effectively brought about by the co-operation of law with medicine. Further, they are likely only to be brought within the sphere of practical politics by agitation initiated by the medical profession, for only medical men know to the full the harm which results from their neglect. If "medical jurisprudence" were interpreted in a wider sense as embracing all the relations in which law and medicine touch each other, the interest of the medical man in the subject would be quickened. He would realise its application to himself and to the community, and would perhaps be induced to take a larger part in the administration of affairs than he does at present. For the training the medical man undergoes should make his views on social matters of peculiar value. His brother of the long robe, who is so much more active in matters of State than he is, sees but a limited side of human nature, and that not usually the best. He is trained to look upon things as fixed or changing but slowly, and to venerate precedent. The physician is necessarily a student of human nature. He sees his fellow-men under every aspect, from the cradle to the grave. He must be an optimist and the disciple of progress. He looks forward rather than backward, and puts no limit to the possibilities of social improvement.

But the fault in the medical curriculum is the absence of studies demanding generalisation. From first to last the attention of the student is focussed upon the individual. Forensic medicine, and to some extent public health, are the only subjects he studies which at all require him to apply his knowledge to the community as a whole. It is this defect which is partially responsible for his lack of interest in public affairs, and it is to remedy it that a fuller training in medical jurisprudence, and a wider definition of its scope, is urged.

The medical man who wishes to study these branches of State medicine must perforce seek his information from many sources. There is no one book which covers the ground, and on many points he must go to the fountain head, the Acts of Parliament themselves. From this point of view, attendances at the coroners' courts, the police-courts and the higher criminal courts will all prove of value. Cases under the Workmen's Compensation Act may be studied in the county courts. From time to time matters are argued in the High Courts involving medical points of great interest and importance. The medical jurist, if unable to attend these cases, should read the reports and endeavour to grasp the legal principles upon which the decisions are based. Of recent years opportunities for the study of legal medicine have been furnished by the existence of the Medico-Legal Society.* Though only founded in 1901, this Society now numbers some 250 members drawn from both the legal and medical professions. Meetings are held monthly at which papers on medico-legal subjects are read and discussed.

A few words may be said with regard to specialising in medical jurisprudence. The greatest opportunity in this direction is afforded by the coroners' service. It is becoming more and more the practice to appoint to important coronerships only men who have qualified in both law and medicine. As a rule these are well paid; the work possesses considerable scientific interest and, though responsible, does not usually involve long hours. The appointments are in the hands of the county councils. Following the recommendations of the Departmental Committee on Coroners' Law and Inquests, which reported some two years ago, it is probable that sooner or later an Act will be passed making some changes in the law regarding coroners. But, whatever may be done, it is not likely that the importance of utilising medical knowledge in the elucidation of deaths from violence will be less recognised. Indeed, it is probable that

* Hon. Secretary, J. Howell Evans, F.R.C.S., 25 Berkeley Square, W. Annual subscription, 10s. 6d.

the influence of the profession will be increased, for the tendency is growing to look upon the coroner's inquest more from the medical than from the legal standpoint. This is shown, among other things, by the inquiries now frequently held into deaths under anæsthetics. Formerly coroners maintained that their chief function was to determine whether or not crime had been committed, and in the evidence given before the Departmental Committee this view was freely expressed. Now that some coroners consider it their duty to inquire into deaths involving highly scientific questions in which there is no suspicion whatever of foul play, the medical profession is entitled to ask that the coroner shall be a medical man, or if not shall in such cases necessarily have the assistance of a skilled medical assessor.

During recent years a certain number of medical men have been called to the Bar. Special knowledge of any description may be of service to a barrister, but it is possible to overrate the value of a medical training in this connection. It will be found useful in a certain number of criminal cases, and rather more frequently in claims under the Workmen's Compensation Act. But the medical barrister will soon discover that in practice he has not the advantage over his colleagues that he may have anticipated. The variety of cases a barrister has to deal with enables him to acquire a power of getting up quickly the special knowledge necessary for any particular case, and if he does much compensation work he soon possesses a knowledge of the special medical and surgical points quite as useful for the purpose as that of the fully qualified medical man.

In some cases medical men have been called to the Bar not with the intention of practising in the courts, but merely to acquire fresh experiences, or to assist other work. Those who can spare the time and expense to do this will find that the task is not a very difficult one, and that their efforts are well repaid. A medical student can easily keep his terms at one of the Inns of Court without interfering with his medical studies. It is only necessary to dine in hall a certain number of days in each term, and twelve terms must so be kept. The examinations in four different branches of law can be taken at any time, and demand far less reading than the examinations for a medical degree. In making this comparison it must be remembered that the medical man is looked upon as competent to practise as soon as he has received his qualification, while a call to the Bar is really little more than a preliminary to legal studies. In the great majority of cases a law student, after taking his call, spends a year or more reading and working in the chambers of a barrister in busy practice, a privilege for which he usually has to pay a good premium. The medical man who becomes called to the Bar, even though he pursue his legal studies no further, will find that new ideas have been opened up to him, and new friends and associations created. The power he will possess of looking at problems from both the legal and the medical standpoints will add to his interest in his work, and will increase his usefulness to the community.

